# Mesenchymal stem cells for regenerative medicine in central nervous system

**DOI:** 10.3389/fnins.2022.1068114

**Published:** 2022-12-13

**Authors:** Man Li, Hong Chen, Mingxin Zhu

**Affiliations:** ^1^Department of Anesthesia, Tongji Hospital, Tongji Medical College, Huazhong University of Science and Technology, Wuhan, China; ^2^Department of Neurosurgery, Tongji Hospital, Tongji Medical College, Huazhong University of Science and Technology, Wuhan, China

**Keywords:** mesenchymal stem cells (MeSH ID D059630), regenerative medicine, central nervous system, immunomodulation, anti-apoptosis

## Abstract

Mesenchymal stem cells (MSCs) are multipotent stem cells, whose paracrine and immunomodulatory potential has made them a promising candidate for central nervous system (CNS) regeneration. Numerous studies have demonstrated that MSCs can promote immunomodulation, anti-apoptosis, and axon re-extension, which restore functional neural circuits. The therapeutic effects of MSCs have consequently been evaluated for application in various CNS diseases including spinal cord injury, cerebral ischemia, and neurodegenerative disease. In this review, we will focus on the research works published in the field of mechanisms and therapeutic effects of MSCs in CNS regeneration.

## Introduction

Injuries and neurodegenerative diseases often bring about loss of neurons and axonal damage to central nervous system (CNS). Neurons fail to regenerate spontaneously in the mature mammalian CNS. Tremendous effort has been devoted to recognizing the mechanism of CNS regenerative failure, yet a complete understanding is still lacking. A broad spectrum of regeneration strategies, particularly by increasing neuronal survival and axon re-extension, have been met with mixed success (Varadarajan et al., 2022).

Mesenchymal stem cells (MSCs) are among the most widely studied multipotent stem cells, which reside in multiple organs and can be derived from various tissues. Their capability of differentiation into almost any end-stage lineage cells and strong paracrine effects make MSCs a promising candidate for endogenous regeneration. Moreover, the MSCs can be transplanted safely and effectively by systemic and local delivery route (Liu et al., 2020). However, the choice of MSC source, including the bone marrow (BM), adipose tissue (AT), and umbilical cord blood (UCB), is critical in determining the therapeutic potential of MSCs (Bortolotti et al., 2015). To date, BM-MSCs and AT-MSCs are the most extensively studied cell sources for CNS repair, because both of them showed similar neuronal differentiation potential (Chung et al., 2013). BM-MSCs can differentiate into astrocytes, neurons and Schwann cell like cells in the peripheral nervous system (PNS) to promote neural regeneration (Tohill and Terenghi, 2004). Meanwhile, some studies have shown that AT-MSCs can secrete various kinds of growth factor, such as brain-derived neurotrophic factor (BDNF), neural growth factor (NGF), and glia cell-line derived neurotropic factor (GDNF), which promotes neuron survival and axonal regeneration (Villoslada et al., 2000; Blesch and Tuszynski, 2003; Kerschensteiner et al., 2003). Compared to BM-MSCs, AT-MSCs produced a significantly larger amount of cytokines and growth factors, which mediate paracrine actions that promote cellular survival pathways and tissue-repair mechanisms (Zhou et al., 2013).

Numerous studies demonstrate that transplantation of MSCs can regulate neuron growth and axon re-extension, and ameliorate nervous system function after CNS injury or degeneration. In this review, we discuss the therapeutic effects of MSCs in CNS regeneration and the potential involved mechanisms.

## Immunomodulation effects of mesenchymal stem cells

**FIGURE 1:**
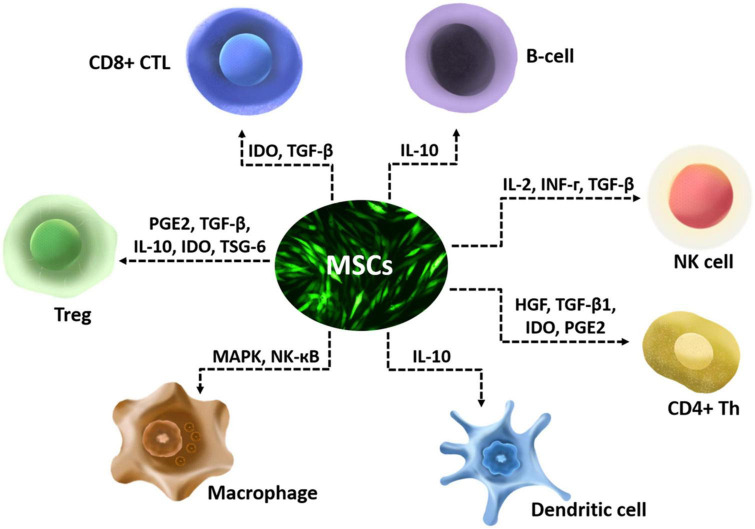
Schematic diagram of immunoregulatory properties of mesenchymal stem cells (MSCs).

The anti-inflammatory effect of MSCs is mostly executed *via* secretion of various enzymes and soluble factors and their paracrine actions on T lymphocytes, including naïve CD4^+^ T-cells, Th1 cells, Th2 Cells, Th17 Cells, CD4^+^ FoxP3^+^ Regulatory T-Cells (Tregs), and CD8^+^ T-cells (Mattar and Bieback, 2015). They also have multiple anti-inflammatory effects that include affecting the chemotactic properties of B cells (Corcione et al., 2006), suppressing interleukin-2 (IL-2) induced natural killer (NK) cell activation (Spaggiari et al., 2006), downregulating NK-activating receptors (Yen et al., 2009), and affect functions of myeloid cells such as monocytes (Jiang et al., 2005), dendritic cells (Ramasamy et al., 2007), and macrophages (Ylöstalo et al., 2012; Figure 1). MSCs modulate immune cells by disrupting their activation, proliferation, maturation, cytolytic activity, cytokine production, or antibody production (Gao et al., 2016). The CNS and its barriers are replete with innate and adaptive immune cells, which interact with glia in diseases. Interactions between immune cells and glia have been shown to perform critical roles in the regenerative capacity of CNS (Greenhalgh et al., 2020). The effects of MSCs on immune cells may participate in the interactions between immune cells and glia, then influence the regeneration of CNS.

Studies on microglia offer further insight into the role of glia and the immune cells in the CNS regeneration since microglia can be defined as both glia and immune cells (Greenhalgh et al., 2020). A recent study has shown that AT-MSCs are able to reprogram microglia/macrophage from a pro-inflammatory M1 phenotype to an anti-inflammatory M2 phenotype (Shao et al., 2020). Zhang et al. (2013) observed that intravenous BM-MSCs transplantation in brain was associated with a lower density of microglia/macrophages and reduced levels of proinflammatory cytokines. Another study that investigated the therapeutic effects of BM-MSCs by systemic transplantation into traumatic brain injury (TBI) model of rats found that MSCs reduced microglia and increased neurogenesis (Kota et al., 2016). Besides, MSCs derived exosomes inhibit microglia inflammatory in the damaged regions in cerebral ischemia models (Zhao et al., 2020).

Extensive data found that MSCs could secrete a variety of soluble molecules include hepatocyte growth factor (HGF), transforming growth factor-β1 (TGF-β1), indoleamine-pyrrole 2,3-dioxygenase (IDO), prostaglandin E2 (PGE2), interleukin (IL)-13, IL-10, IL-12p70, IL-17E, and IL-27 to exert anti-inflammatory potential (Ryan et al., 2007; Ren et al., 2008; Sobacchi et al., 2017). Human MSCs isolated from BM, AT, dental pulp, Wharton’s jelly (WJ) and placenta paracrine anti-inflammatory factors, such as TGF-β, to promote neuroprotective effects (Ryan et al., 2007; Tomic et al., 2011; Zhou et al., 2011; Carrillo-Galvez et al., 2015; Heo et al., 2016). And the production of TGF-β by MSCs can be increased by proinflammatory cytokines, substrate rigidity, glucose levels and hypoxia (de Araujo Farias et al., 2018). On the other hand, TGF-β has also been shown to signal *via* SMAD2/3 phosphorylation in MSCs to regulate the biology of MSCs themselves (Choy and Derynck, 2003). By the ability to secrete bioactive and trophic factors, MSCs exert a significant influence on cellular regeneration and new tissue growth (Scuteri et al., 2011).

Although all the MSCs share basic properties, there are subtle differences among MSC types that may affect their immunomodulation. A recent study that compared the immunomodulatory effects of MSCs derived from BM, AT, and WJ of the umbilical cord on T-lymphocytes by co-culture, found that AT-MSCs showed the strongest effect on downregulating CD38 expression on activated T-lymphocytes, whereas BM-MSCs had the weakest effect (Najar et al., 2010). Meanwhile, Ribeiro et al. (2013) found that AT-MSCs emerged as the most immunosuppressive population, as hamper T-cell proliferation by arresting them in the non-activated compartment. However, another research demonstrated that AT-MSCs and BM-MSCs showed equal ability to induce Th0 differentiation into Th1 and Th2 (Xishan et al., 2013). Meanwhile, in a mouse model study, UCB derived MSCs and BM-MSCs showed a similar inhibition of Th17 cells (Li et al., 2013). Although the precise mechanism of these anti-inflammatory effects remains unclear, current clinical trials show that intravenous administration of MSC is a safe and effective treatment for immune disease (Li et al., 2021).

## Anti-apoptotic effects of mesenchymal stem cells

An *in vitro* study showed that BM-MSC could modulate neuronal and glial response to apoptosis in amyotrophic lateral sclerosis (ALS) (Sun et al., 2013). Other studies also demonstrated that intracerebral (Kim K. et al., 2015; Zhou et al., 2016), intravenous (Wang et al., 2012; Chen et al., 2015), or intracerebroventricular (Park W. S. et al., 2016) transplantation of MSCs could ameliorate apoptosis of endogenous neural cells. The infiltrated inflammation-associated immune cells released numerous reactive oxygen species that led to programmed cell death in the injured area. MSCs may suppress oxidative stress and increase the anti-apoptotic Bcl-2 gene expression in brain (Gu et al., 2014). Previous studies showed that miRNA including miR-134 (Xiao et al., 2019), miR-138-5p (Deng et al., 2019), miR132-3p (Pan et al., 2020), miR-21-3p (Li et al., 2019), and miR-22-3p (Zhang et al., 2021) play important roles in these MSCs mediated anti-apoptosis effects in brain. miR-22-3p derived from AT-MSCs prevents neuron apoptosis by inhibiting KDM6B mediated BMP2/BMF axis (Zhang et al., 2021). These effects were abolished by inhibition of miR-22-3p. After intravenous transplantation, AT-MSCs inhibit neural apoptosis by reducing the abnormally high level of miR-21-3p in middle cerebral artery occlusion rat models (Li et al., 2019). It is demonstrated that miR-21-3p directly inhibits the MAT2B expression in neural cells, and miR-21-3p inhibition in neurons attenuated hypoxia/reoxygenation induced impairments. Meanwhile, BM-MSCs deliver anti-apoptotic miRNAs to protect oligodendrocytes, astrocytes, and endothelial cells from apoptosis, which facilitate axon re-extension (Deng et al., 2019; Xiao et al., 2019; Pan et al., 2020).

## Axon re-extension effects of mesenchymal stem cells

Axon regeneration after injury is defined as axon regrowth and the subsequent innervation of injured region, resulting in recovery of function to the CNS. Axon re-extension is defined as axon lengthy regrowth that carry out *de novo* growth over long distances to reach their targets. It has been widely accepted that many extrinsic factors derived from the external environment around damaged areas limit axonal re-extension, such as chondroitin sulfate proteoglycan (CSPG) (Shen et al., 2009), myelin-associated glycoprotein (MAG) (Hasegawa et al., 2004), oligodendrocyte-myelin glycoprotein (von Büdingen et al., 2015), and Nogo-A (Schwab and Strittmatter, 2014). Preventing these inhibitory signals has been considered as a promising approach to promote axon re-extension.

Mesenchymal stem cells have been demonstrated to help neurites to overcome the inhibitory effects of Nogo-A, MAG, and CSPG. In MSC/neuronal cocultures, MSCs promote spinal neuronal adhesion and neurite extension over Nogo-A and MAG (Wright et al., 2014). miR17-92 derived from MSCs overcome the inhibitory effect of CSPGs, when cultured together (Zhang et al., 2017). In spinal cord injury (SCI) dogs, induced using compression method, local transplanted AT-MSCs prevent the accumulation of CSPG and enhance axonal extension (Park et al., 2012). In addition to inhibiting of the extrinsic factors, MSCs provides a favorable microenvironment for re-establishment of functional local circuits with HGF, epidermal growth factor (EGF), neurotrophin-3 (NT-3), and GDNF (Bai et al., 2012; Lv et al., 2021).

## The potential role of mesenchymal stem cells in central nervous system regeneration

Mesenchymal stem cells originate from BM, AT, UCB, and synovium are capable of differentiation along mesodermal lineages other than that of their tissue of origin, so they were investigated mostly in clinical (Dawn and Bolli, 2005). MSC expression of neuronal or astrocytic marker has been observed *in vitro* (Fesharaki et al., 2018) and *in vivo* (Ma et al., 2018). Meanwhile it is generally accepted that MSCs can secrete several growth factors, such as BDNF, NGF, vascular endothelial growth factor (VEGF), GDNF and insulin-like growth factor 1 (IGF-1), which can facilitate neurogenesis, and create a favorable microenvironment for re-extension and remyelination during reconstruction to play a crucial role in nourishing and protecting neurons (Zhang et al., 2004; Vercelli et al., 2008; Uccelli et al., 2011; Muto et al., 2012). So MSCs have been widely studied and applied in regenerative medicine in nervous system. In this section, we summarize reports concerning the latest preclinical and clinical trials of various MSC types for tissue engineering in CNS. In the area of CNS regeneration, MSC based therapy mainly focuses on damage of CNS caused by severe trauma and continuous ischemia and CNS dysfunction caused by neurologic disease.

### Spinal cord injury

Spinal cord injury results in immediate loss of nervous tissue followed by permanent deficits in sensory and motor functions below the injured spinal cord segment. The common promising experimental therapies for SCI include neurotrophic factors, enzymes and antibodies against inhibitory molecules, activated macrophages, bridging scaffolds and stem cell transplantation. The therapeutic approach differs depending on the stage after SCI. Traumatic SCI can be divided into acute phase, subacute phase, and chronic phase. The acute phase of SCI starts after injury and persists for hours to days. The acute phase involves the release of excitotoxicity, the breakdown of the blood-brain barrier, localized edema, and accelerated apoptosis (Emery et al., 1998). The chronic phase of SCI is associated with local inflammation, apoptosis, and ongoing demyelination (Schwab and Bartholdi, 1996; Fleming et al., 2006). Since most SCI patients remains in chronic phase, this phase attracts the greatest research interest among scientists and doctors. In animal models of SCI, stem cell-based regenerative approach has been demonstrated to elicit anatomical repair often accompanied by functional recovery (Ritfeld et al., 2012; Forraz et al., 2013). Stem cell-based regenerative medicine has become a new promising therapeutic approach for treating SCI (Lv et al., 2021; Nakazaki et al., 2021).

Mesenchymal stem cells have the potential to create a reparative environment, which is the main motivation for exploring MSCs for regenerative medicine in nervous system (Yang et al., 2008; Caplan, 2009). *In vivo* experiments employing different SCI models and various routes of MSCs administration revealed significant functional recovery. After transplantation of human WJ-MSCs into lesion site of complete spinal cord transection rats, the numbers of regenerated axons in the corticospinal trace and neurofilament positive fibers around the lesion site were increased (Yang et al., 2008). It was also reported that intraspinal grafting of rats BM-MSCs into the construction injured spinal cord promotes axonal regrowth and reduces the lesion volume (Gu et al., 2010). Meanwhile, MCSs that overexpress some molecules, such as NT-3 (Stewart et al., 2018), IL-10 (Gao et al., 2022), IL-13 (Dooley et al., 2016), and hemeoxygenase-1 (Khan et al., 2019), can elicit improved axon regeneration and promote motor functional recovery in SCI models.

Since AT-MSCs produced a significantly larger number of cytokines and growth factors than BM-MSCs, some publications suggest AT-MSCs to be an alternative to BM-MSCs for the cellular therapy of SCI (Forostyak et al., 2013). However, while AT-MSCs have been evaluated in animal SCI models, there remains a paucity of large and longitudinal clinical trials. The obstacles for clinical translation of MSCs are the low engraftment and poor survival (Qin and Zhao, 2020), and whether the MSCs can really provide benefit to patients (Staff et al., 2019). El-Kheir et al. (2014) conducted a phase I/II controlled single-blind clinical trial, in which SCI patients received an intrathecal injection of autologous BM-MSC combined with physical therapy showed functional improvements and no long-term cell therapy related side effects over patients received physical therapy alone. Vaquero et al. conducted a phase I, single center, non-randomized, uncontrolled clinical trial in span (NCT02165904). This study evaluated the effects and safety of the subarachnoid transplantation of autologous BM-MSC in patients with chronic SCI reported that most patients showed sensitivity improvement using American Spinal Injury Association score, and BM-MSC was associated with bronchitis in one patient.

### Cerebral ischemia

Ischemic stroke induces an extensive neuro-inflammatory response, which seems to be responsible for the propagation of brain damage. However, experimental therapies aimed at reducing immunological reactions after ischemic stroke using cell inhibitors or mediators have not been successful. In this situation, new therapeutic strategies using stem cells have emerged as a promising tool. The most frequently used stem cells are the MSCs, because of their great trophic capabilities (Laso-Garcia et al., 2019). The possible mechanisms involved in potential therapeutic activity of MSCs including neuroprotection, immunomodulation, and activation of neurogenesis, synaptogenesis, astrogenesis, oligodendrogenesis, and angiogenesis in stroke (Dabrowska et al., 2019). Current research suggests that the beneficial effects exerted by MSCs are mainly related to differentiation and immune modulatory mechanism (Zachar et al., 2016).

Chen et al. (2001) reported that BM-MSC transplanted rats showed significant recovery in somatosensory behavior and neurological severity score after cerebral ischemia. Rat WJ-MSCs were shown to have a protective action when transplanted 3 days before a cardiac arrest induced global ischemia by an extracellular signaling mechanism (Jomura et al., 2007). This recovery was accompanied by a decrease in inflammatory reaction after global ischemia. When transplanted with human UCB-MSCs, cerebral ischemia animals presented reduced lesion size and higher extent of vascularization in ischemic areas. Meanwhile, the expression of SDF-1, BDNF, and GNF was higher in ischemic tissues following MSCs treatment (Ding et al., 2007). The authors of a 2018 meta-analysis concluded, “in preclinical studies, Median quality score 4.90/10; confidence interval 95% and large effect size were observed, that strongly supports the translation potential of MSCs therapy for ischemic stroke (Sarmah et al., 2018).”

In a non-randomized small trial with BM-MSCs, the authors found improvements in clinical outcome (European Stroke Scale, National Institutes of Health Stroke Scale, and Fugl-Meyer total score) with stroke patients (Steinberg et al., 2016). Meanwhile Levy et al. (2019) reported that intravenous transfusion of allogenetic MSCs in patients with chronic stroke suggested behavioral gains in a randomized, placebo-controlled study. Another phase 1 clinical trial also demonstrated the safeness of intravenous BM-MSCs use for cerebral ischemia in human (Vahidy et al., 2019). However, all the clinical trials were small trials, so their results should be taken with caution.

### Neurodegenerative diseases

The increasing prevalence of CNS disorders has been attributed to neurodegenerative diseases including Alzheimer’s disease (AD), Parkinson’s disease (PD), Huntington’s disease (HD), amyotrophic lateral sclerosis (ALS), multiple sclerosis (MS), and multiple system atrophy (MSA) (Przedborski et al., 2003). A common characteristic among such disorders is progressive neuronal death that leads to debilitating neurologic impairments. Although our understanding of the neurodegenerative disease pathology has been improved these years, a precise and reliable treatment has not been accomplished. Current common treatments just relieve symptoms without affecting the major pathological characteristics of these diseases. MSCs hold great potential for cell therapy as they can differentiate toward neural fates and secrete a broad range of factors, which are able to promote neuroprotective or regenerative mechanisms. Moreover, upon transplantation, MSCs possess the capability to home toward neural lesions, implying their potential use as vehicles for therapeutic agents administration (Volkman and Offen, 2017).

Mesenchymal stem cells transplantation often improved survival rates, declined pathology, and rescued cognitive function decline in multiple rodent models of neurodegenerative diseases (Volkman and Offen, 2017). Preclinical studies found that MSCs from BM (Babaei et al., 2012), AT (Kim et al., 2012), UCB (Lee H. J. et al., 2012), and the placenta (Yun et al., 2013) have the ability to regulate amyloid pathology through neuroinflammation, which plays a crucial role in the progression of several neurodegenerative diseases. Bayat et al. (2021) demonstrated that intracerebral transplantation of human olfactory ecto derived MSCs could promote behavioral and anatomical recovery in a HD rat model. Study on conditioned medium of human amniotic membrane derived MSCs found that intraperitoneally injection of this conditioned medium could significantly decrease microglia activation in the R6/2 HD mouse model (Giampa et al., 2019). It is also demonstrated that human UCB-MSCs decreased secretion levels of the proinflammatory cytokines TNF-α and IL-1β, and increased level of the anti-inflammatory markers of IL-4, AMCase, YM-1, and Arg-1 in an AD mouse model (Lee H. J. et al., 2012). Following work showed that human BM-MSCs promote secretion of IL-4 from microglia cells and stimulated α-synuclein clearance in a PD mouse model (Park H. J. et al., 2016). Fontanilla et al. (2015) found that NGF might be responsible for the effects of AT-MSCs in SOD1 G93A mice, defined as the preservation of motor neurons and inflammatory pathway inhibition. To enhance their typical trophic support, MSCs have been genetically engineered to overexpress neurotropic factors, such as NGF, BDNF, and GDNF, whose neuroprotective actions are widely acknowledged (Lo Furno et al., 2018). Meanwhile, BDNF engineered MSCs have been considered for studies of regeneration in ALS, AD, PD, and HD, and even in SCI, TBI, and peripheral nerve injury (Deng et al., 2016).

Clinical studies indicated MSC-based therapy as a safe and feasible technique for patients with AD (Kim H. J. et al., 2015), PD (Venkatesh and Sen, 2017), ALS (Sykova et al., 2017), and MSA (Lee P. H. et al., 2012). Since preclinical and clinical studies have demonstrated the effectiveness of MSCs for the treatment of neurodegenerative disease, many researches begin to focus on the method to enhance the effects.

### Other central nervous system disease

Although the clinical application of MSCs therapy in CNS disease currently remains infancy, MSCs research has rapidly expanded over the past decade. Besides neurodegenerative diseases, cerebral ischemia, and SCI, numerous animal model studies have also demonstrated the effects of MSCs in epilepsy (Agadi and Shetty, 2015). BM-MSCs can reduce epileptogenesis by inhibiting neuronal cell death and suppressing aberrant mossy fiber sprouting in a rat model of epilepsy (Fukumura et al., 2018). UCB-MSCs might enhance GABA neurotransmitter levels and ameliorate oxidative stress damage in pentylenetetrazole-induced chronic epilepsy in rats (Mohammed et al., 2014). Moreover, in a phase 1 open label study, MSCs can be a safe and promising candidate for cell therapy in anti-epileptic drugs resistant epilepsy patients (Hlebokazov et al., 2017). To improve the therapeutic effect of MSCs in a mouse model of epilepsy, genetically engineered MSCs, such as IL-13 engineered MSCs, which showed enhanced neuroprotective and disease-modifying effects, has been used (Ali et al., 2017).

### Approaches to enhance therapeutic effects of mesenchymal stem cells

Although MSCs represent a promising candidate for CNS regeneration, low therapeutic efficacy limits their clinical use. Different culture conditions may result in altered survival, homing, and key functional features of MSCs. Madrigal et al. (2014) found that cell culture under hypoxic conditions has potential effects on MSCs therapeutic property by increasing the secretion of HGF, TGF-b, VEGF, TSG-6, which is important in CNS regeneration. Others demonstrated that pro-inflammatory stimuli and tri-dimensional growth stimulate trophic factors secretion of MSCs (Vizoso et al., 2017). It is evident that culture conditions will considerably affect the therapeutic efficacy of MSCs. Apart from culture medium, developed therapeutic strategies may also enhance therapeutic effects of MSCs such as delivery route and timing. Although there is no consensus on the optimum delivery route of MSCs, intracerebroventricular transplantation may be the most efficacious. By reviewing previous pre-clinical and clinical studies, Park et al. (2018) found that intracerebroventricular transplantation of MSCs may be associated with enhancement of endogenous, compared to intravenous and intraparenchymal routes for CNS regeneration. The intracerebroventricular transplanted MSCs attenuated brain injury in a time-dependent manner. Significant neuroprotection was demonstrated when administered from 2 to 7 days after induction in intraventricular hemorrhage rat models (Park H. J. et al., 2016).

## Conclusion

Mounting evidence suggests that MSCs can be a potential therapy to promote CNS regeneration and functional restoration. The therapeutic role of MSCs is extremely complex. Demonstrating their exact interaction with other cells during neuronal survival, axon re-extension, synapse re-formation, and re-myelination may help researchers to optimize the effects of MSCs based therapies. Optimal conditioned culture, delivery route, and timing of MSCs may be a promising strategy to improve therapeutic effects. In conclusion, study MSCs in CNS provides insight into the exact mechanism of CNS regeneration and repair, helps optimize cell based therapy.

## Author contributions

ML wrote the manuscript and polished it up for publication. HC created the figures. MZ gave advice and edits. All authors contributed to the article and approved the submitted version.
